# Long-term outcome after SphinKeeper® surgery for treating fecal incontinence—who are good candidates?

**DOI:** 10.1007/s00423-023-03188-6

**Published:** 2023-12-06

**Authors:** Christopher Dawoud, Kerstin Melanie Widmann, David Pereyra, Felix Harpain, Stefan Riss

**Affiliations:** Department of General Surgery, Division of Visceral Surgery, Medical University Vienna, Waehringer Guertel 18-20, 1090 Vienna, Austria

**Keywords:** Fecal incontinence, SphinKeeper®, Prostheses dislocation, Prostheses migration

## Abstract

**Purpose:**

The efficacy of the novel SphinKeeper® procedure for the treatment of fecal incontinence (FI) is not yet well defined. This study aimed to assess long-term functional outcomes after SphinKeeper® surgery.

**Methods:**

We included 32 patients with FI (28 female), who were operated at a tertiary referral center between August 2018 and September 2021. Functional outcome and quality of life were evaluated prospectively using validated questionnaires before and after surgery. Additionally, endoanal ultrasound and anal manometry were conducted prior and after SphinKeeper® implantation. Predictive parameters for treatment success were defined.

**Results:**

The mean follow-up time was 22.62 ± 8.82 months. The St. Mark’s incontinence score decreased significantly after surgery (median preoperative = 19 (IQR 17–22) versus median last follow-up = 12 (IQR 8–16), *p* = 0.001). Similarly, physical short-form health survey showed a significant improvement after SphinKeeper® implantation (*p* = 0.011).

Patients with a higher degree of internal sphincter defect showed an improved objective therapy success (*r* = 0.633, *p* = 0.015) after SphinKeeper® operation, whereas the type and severity of FI had no impact on the functional outcome. Notably, a higher number of dislocated prostheses (*r* = 0.772, *p* = 0.015) showed a significant correlation with reduced improvement of incontinence.

**Conclusion:**

The SphinKeeper® procedure showed a significant long-term functional improvement in over half of the patients. Patients with a higher internal sphincter defect benefited most, whereas dislocation of the prostheses was associated with less favorable results.

## Introduction

Fecal incontinence (FI) represents a devastating condition with a prevalence ranging from 1 to 20% in the general population [1–3]. The wide range can be explained by the heterogeneity of available studies and by its taboo topic.

If conservative treatment fails, surgical therapy can improve patients’ complaints and quality of life. Although FI occurs frequently, the number of potential operations is limited and often associated with disappointing outcomes. Surgical procedures comprise the injection of bulking agents, sphincteroplasty or, most effectively, sacral neuromodulation.

The GateKeeper® was introduced as a novel operation defined by the implantation of up to 4 solid prostheses into the intersphincteric grove in 2011 [4]. Early data were promising, showing success rates of above 50% with only a low number of perioperative complications [5]. Subsequently, the technique was further modified by using slightly longer and more (up to 10) prostheses and was renamed SphinKeeper®. Recent short-term publications described a significant improvement in incontinence episodes and quality of life [6–11].

Notably, several studies described a migration and dislocation tendency of implanted prostheses. Ratto et al. included ten patients and found a dislocation of a single prosthesis in only one patient [6]. Another investigation by Trenti et al. found a migration rate in 51% of patients using the GateKeeper® with a higher number of migrated prostheses in the non-responder group [12].

The purpose of this long-term cohort study was to evaluate the clinical success rate of SphinKeeper® implantation. In addition, predictive parameters for treatment success were analyzed.

## Methods

Thirty-two patients with FI, who underwent SphinKeeper® procedure at a single tertiary referral center, were enrolled prospectively.

The local Ethics Committee of the Medical University of Vienna granted approval. This study was registered at ClinicalTrials.gov (NCT04992429).

Patients were included, who were older than 18 years and reported incontinence for liquid and/or solid stool and flatus incontinence for at least 6 months. All patients were refractory to conservative management including diet changes, stool regulative medication, pelvic floor exercises, and biofeedback therapy for 3 months. Exclusion criteria were local malignant diseases, inflammatory bowel disease, chronic diarrhea without responsiveness to medical treatment and anal fistula disease. A sphincter defect was not regarded as a contraindication for SphinKeeper® therapy.

Patients received an endoanal ultrasound examination (EAUS; Flex Focus 500, BK Medical Holding Company, Inc.) to assess internal anal sphincter (IAS) and external anal sphincter (EAS) configurations. Anorectal manometry (ARM) was conducted (THD® Anopress, Pressprobe, Sensyprobe) to analyze resting, squeezing and straining pressure. Anorectal compliance and sensations were recorded by the balloon test.

Functional outcome was assessed using validated standardized questionnaires before surgery, after 3, 6 months and at the last follow-up appointment. The St. Mark’s incontinence score was used to measure FI [13], and obstructive defecation syndrome (ODS) was evaluated by the Wexner constipation score [14]. Quality of life was measured by the short-form health survey (SF-12) [15].

### Surgical technique

The surgical implantation was performed in the lithotomy position as described previously [8]. All operations were conducted or supervised by one colorectal surgeon. Patients received a preoperative enema (Klistier® Fressenius, 130 ml) and a single-antibiotic prophylaxis (cefuroxime 1.5 g and metronidazole 1.5 g). In addition, a urinary catheter was installed to allow patients to maintain 24 h of bed rest postoperatively. Subsequently, 9–10 2-mm skin incisions were made at a 2–3 cm distance from the anus. The intersphincteric space was then entered using the delivery system, and the position was checked by endoanal ultrasound. The same procedure was repeated for all ten prostheses around the entire circumference. The skin wounds were closed with absorbable sutures.

### Outcome measurements

The primary outcome was defined as a change of FI assessed by St. Mark’s incontinence score at each follow-up visit. In order to evaluate the objective treatment effect and to quantify the dynamic of FI severity, the delta of St. Mark’s incontinence score from prior to SphinKeeper® implantation to the last follow-up was calculated (deltaSMS). Of note, an amelioration of FI severity over time is expressed as a negative value for deltaSMS.

Secondary, dislocation and migration of the implants were recorded, and their impact on functional outcomes was analyzed. The position was considered correct if more than 50% of the prostheses showed correct placement (no dislocation or migration) at the same vertical level (at least two-thirds of the prostheses within the target area as reported by Litta et al. [11]).

The movement of each prothesis along the intersphincteric space was defined as dislocation, the movement through the sphincteric muscle or out of the intersphincteric space as migration.

### Statistical analysis

Statistical analysis was performed using the SPSS statistical software package (IBM SPSS Statistics for Mac, Version 26.0). For descriptive statistics, the Shapiro–Wilk test was applied to evaluate normal distribution of the data. Whenever a normal distribution was identified, the mean and standard deviation were reported. Otherwise, the range and the median were mentioned. Accordingly, Student’s *t*-test or the Wilcoxon signed rank tests were applied as appropriate. Consequently, non-parametric tests were used for the exploratory statistics due to the sample size of *n* = 32. Here, continuous variables are expressed as median and interquartile range (IQR). Categorical variables are presented as numbers with percentages. Quantitative variables were compared using the Mann–Whitney U test or Wilcoxon test. To explore dichotomous variables, chi-squared test was used. A *p*-value < 0.05 was considered to denote statistical significance.

## Results

### Patient characteristics

Between August 2018 and September 2021, 32 patients (28 women and 4 men) suffering from fecal incontinence were treated using the SphinKeeper®. The mean follow-up time was 22.62 ± 8.82 months. Patients’ characteristics are outlined in Table 1.

**Table 1:** The demographic and baseline characteristics of included patients

	*n* = 32
Demographics	
Age (years), mean ± sd	72.75 ± 10.66
Female sex, *n* (%)	28 (87.5)
BMI (kg/m^2^), mean ± sd	26.95 ± 5.65
Clinical history	
History of smoking *n* (%)	6 (18.8)
Childbirth, *n* (%)	18 (56.3)
No perineal tear, *n* (%)	12 (37.5)
Perineal tear, grade II, *n* (%)	6 (18.8)
Perineal tear, grade III, *n* (%)	3 (9.1)
Perineal tear, grade IV, *n* (%)	1 (3.1)
Main cause of Fi	
Iatrogenic sphincter injury, n (%) Idiopathic sphincter injury, n (%) Obstetric sphincter injury, n (%)	12 (37.5) 16 (50.0) 4 (12.5)
Previous pelvic floor surgery, *n* (%) Hysterectomy, *n* (%) Sphincteroplasty surgery, *n* (%) Rectopexy, *n* (%) Sacral nerve stimulation, *n* (%)	26 (81.3) 12 (37.5) 2 (6.2) 5 (15.6) 7 (21.9)
Internal sphincter defect, *n* (%) data missing, n (%)	18 (56.3) 3 (9.4)
External sphincter defect, *n* (%) data missing, n (%)	16 (50.0) 3 (9.4)
FI form	
Active FI, *n* (%)	20 (62.5)
Passive FI, *n* (%)	4 (12.5)
Mixed FI, *n* (%)	8 (25.0)
Duration of FI until surgery (months), median (IQR)	56 (28.0–88.5)

*BMI* body mass index

### Surgical outcome

The median number of implanted prostheses was 10 (IQR 9–10), and the median operative time was 37.5 min (IQR 34–48.75). The median postoperative length of hospital stay for patients was 2 days, with an interquartile range of 2–3.

No intraoperative or in-hospital complications were reported. One patient reported perianal discomfort due to the subcutaneous migration of two prostheses after 3 months postoperatively. Those two very superficial located protheses were explanted uneventfully, resulting in resolving of his complaints. This patient experienced functional impairment following the removal and was subsequently subjected to a Re-Do SphinKeeper® implantation using two prostheses [16].

### Functional outcome

**Fig. 1:**
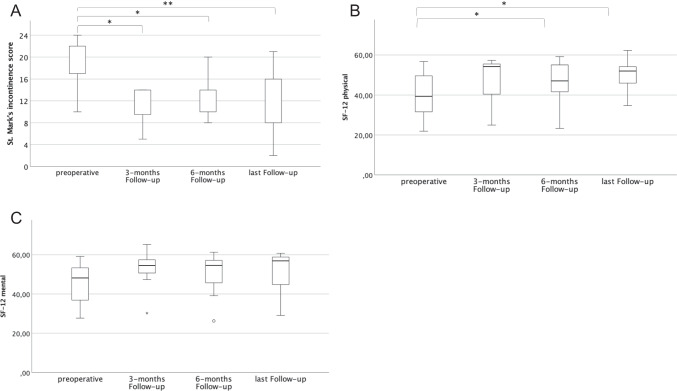
The course of parameters from preoperative to 3, 6, and the last follow-up visit. **A** St. Mark’s incontinence score, **B** Physical SF-12, **C** Mental SF-12. *p < 0.05; **p < 0.005

The St. Mark’s incontinence score decreased significantly comparing preoperative values to the evaluation at last follow-up (median preoperative = 19 (IQR 17–22) versus median last follow-up = 12 (IQR 8–16), *p* = 0.001; Fig. 1A), demonstrating an amelioration of FI after SphinKeeper® implantation. Similarly, physical SF-12 as an indicator of quality of life significantly improved during the follow-up (median preoperative = 39.4 (IQR 28.6–49.7) versus median last follow-up = 52.0 (IQR 45.9–54.2), *p* = 0.011; Fig. 1B). In contrast, the SF-12’s mental component did not show a significant difference (median preoperative = 48.2 (IQR 36.7–54.6) versus median last follow-up = 56.9 (IQR 44.6–58.8), *p* = 0.893; Fig. 1C). The Wexner constipation score at the last follow-up did not differ significantly from preoperative controls (data not shown).

In addition, subjective therapy response was evaluated at the last follow-up visit. Here, 53.8% of patients reported having experienced a symptom relief of at least 50%.

Regarding anal manometry, no significant dynamics were observed after surgery (Table 2). Furthermore, the evaluation of anal sensitivity using the balloon test was comparable between each visit (Table 2).

**Table 2:** The results of anorectal manometry (mmHg) and sensitivity (ml) before and after surgery using Anopress® and Sensyprobe®

*n* = 32	Preoperative	Follow-up 3 months	Follow-up 6 months	Last follow-up	p*
Anorectal manometry					
Resting pressure (mmHg), mean ± sd	22.1 ± 11.7	25.5 ± 13.2	22.7 ± 8.3	25.4 ± 17.6	0.109
Squeezing pressure (mmHg), mean ± sd	42.9 ± 24.1	50.2 ± 33.5	46.7 ± 19.1	50.8 ± 26.3	0.715
Maximal squeezing pressure (mmHg), mean ± sd	57.3 ± 36.8	-	-	58.3 ± 31.4	0.173
Straining pressure (mmHg), mean ± sd	34.1 ± 23.1	33.9 ± 14.3	38.5 ± 17.8	37.6 ± 20.8	0.273
Anorectal sensitivity					
First anorectal sensation (ml), mean ± sd	76.3 ± 77.1	26.7 ± 14.1	26.8 ± 13.2	30.0 ± 19.1	0.269
Rectal tenesmus (ml), mean ± sd	111.1 ± 67.3	59.4 ± 18.1	56.5 ± 23.2	70.0 ± 42.6	0.144
Maximal tolerated volume (ml), mean ± sd	150.0 ± 76.3	105.0 ± 45.7	93.5 ± 36.9	120.0 ± 75.6	0.285

*p = the Wilcoxon test for connected samples between the mean of the preoperative results and of the last follow-up examination

### Prostheses displacement and treatment success

**Fig. 2:**
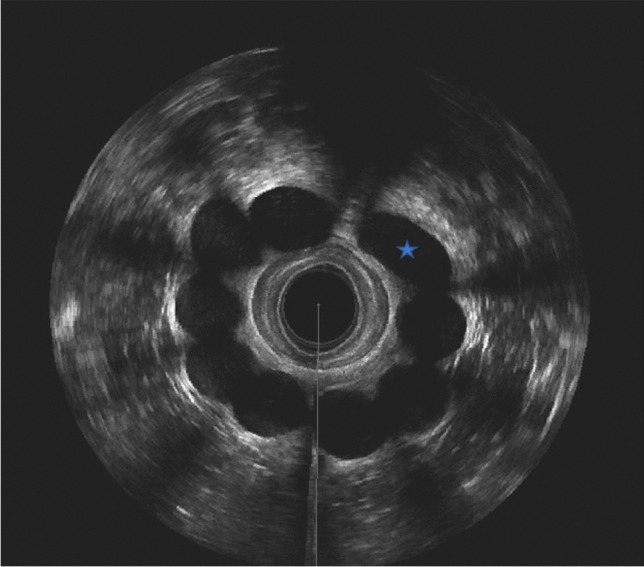
In the performed endoanal ultrasound, 9 prostheses are visible in correct localization in the intersphincteric space. However, one prosthesis is tilted (*) and thus classified as dislocated

The occurrence of prostheses migration and dislocation is further described in Table 3. During their most recent follow-up visit, 14 patients (43.8%) had dislocated prostheses, and 13 patients (40.6%) had migrated prostheses (at least one). In 20 patients, more than 50% of the prostheses showed correct placement, thus located inside the targeted area (Fig. 2).

**Table 3:** The prostheses location at 6 months and at the last follow-up after SphinKeeper® implantation

*n* = 32	Follow-up 6 months	Last follow-up
	*	**
Prostheses evaluation		
Patients with correctly placed prostheses, *n* (%)	14 (43.8)	20 (62.5)
Number of correctly placed prostheses, median (IQR)	6 (5.5–7)	6.5 (6–7.75)
Patients with dislocated prostheses, *n* (%)	14 (43.8)	14 (43.8)
Number of dislocated prostheses, median (IQR)	1 (0–2)	1 (1–2)
Patients with migrated prostheses, *n* (%)	18 (56.3)	13 (40.6)
Number of migrated prostheses, median (IQR)	1 (1–3)	1 (1–3.75)

Correctly placed prostheses were defined as more than 6 prostheses at the same vertical level, at the level of the upper and middle thirds of the anal canal and inside the intersphincteric space. The movement of each prothesis along the intersphincteric space was defined as dislocation, the movement through the sphincteric muscle or out of the intersphincteric space as migration.

*missing data: *n* = 11; **missing data: *n* = 5.

### Determination of treatment success

In regards to objective treatment response, deltaSMS was significantly lower in patients who reported a subjective treatment response at last follow-up (median no subjective response =  − 3.0, median subjective response =  − 8.0, *p* = 0.005).

In order to evaluate whether patients with severe FI might eventually display an increased benefit from SphinKeeper® operation, the cohort was further divided according to the preoperative St. Mark’s incontinence score (cut-off at 12 points). No difference in deltaSMS was observed between patients with high or low preoperative St. Mark’s incontinence scores (median low =  − 6.5, median high =  − 8.0, *p* = 0.958).

Next, the effect in association with the type of FI was evaluated. Here, no differences were observed between patients with active, passive or mixed FI (median active =  − 6.0, median passive =  − 7.5, median mixed =  − 4.5; active vs passive: *p* = 0.641, active vs mixed: *p* = 0.733, passive vs mixed: *p* = 1.000).

**Fig. 3:**
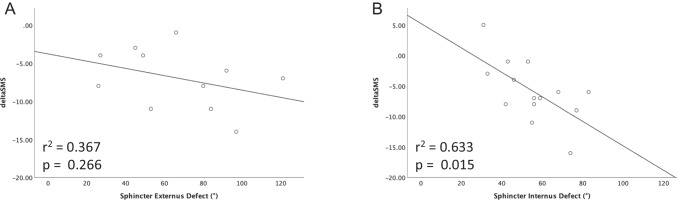
The correlation between the external sphincter damage (3A) respectively the significant correlation of the internal sphincter damage (3B) in degrees and deltaSMS

No correlation was observed between external sphincter damage in degrees and deltaSMS (*r* =  − 0.367, *p* = 0.266; Fig. 3A). However, the degree of damage of internal sphincter defects showed a strong correlation with deltaSMS (*r* =  − 0.633, *p* = 0.015), indicating an improved outcome specifically for patients with high degrees of internal sphincter damage (Fig. 3B).

Lastly, the cohort was divided according to respective cut-offs for parameters gathered via rectal manometric examination. Patients with a low preoperative resting sphincter pressure showed a non-significant numeric trend towards improved deltaSMS over time of follow-up when compared with patients with high resting pressure at baseline (cut-off = 22 mmHg; median low resting pressure =  − 6.0, median high resting pressure =  − 3.0, *p* = 0.310).

A similar pattern was observed for patients with high or low squeezing pressures in anorectal manometric tests (cut-off = 34 mmHg, median low squeezing pressure =  − 6.0, median high squeezing pressure =  − 3.0, *p* = 0.667). In regards to straining pressure, a median at 26 mmHg was used as cut-off. Here, patients with a low straining pressure displayed a trend towards improved deltaSMS, which did not reach statistical significance (median low straining pressure =  − 7.0, median high straining pressure =  − 2.0, *p* = 0.114).

**Fig. 4:**
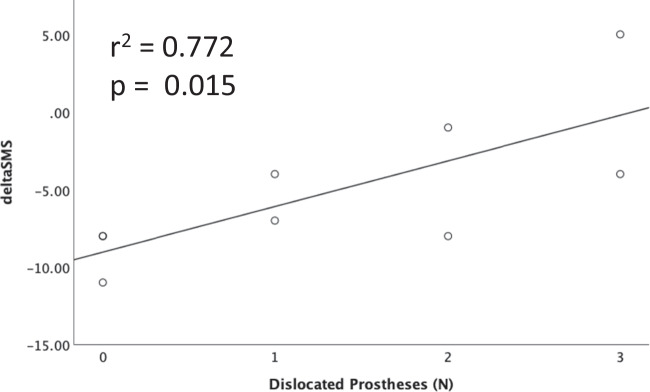
The negative correlation of the number of dislocated prostheses with deltaSMS

The number of dislocated prostheses (*r* = 0.772, *p* = 0.015) showed a significant correlation with subjective treatment response in terms of deltaSMS until last follow-up, which indicates the relevance of correct placement of prostheses (Fig. 4).

## Discussion

The current analysis revealed a significant improvement of functional parameters and quality of life after the SphinKeeper® surgery. Notably, no effect on anorectal manometry dynamics could be detected. In addition, we were able to define parameters for selecting appropriate candidates for this new device.

The SphinKeeper® device has been the subject of a few studies, whereby most of them reported favorable results with significant improvements of clinical symptoms similar to our findings [6–11, 17]. All of them showed a safe implantation with hardly any short- and long-term complications.

La Torre et al. assessed 13 patients, who received SphinKeeper® treatment and measured the anal manometric resting and squeeze pressures, the Cleveland Clinic Fecal Incontinence Score (CCFIS), and the Fecal Incontinence Quality of Life scale (FIQOL) before and 6 months after surgery [9]. They showed that after the procedure, the total number of FI episodes per week and the anal maximal resting pressure improved significantly. Noteworthy, 61.5% of comprised patients had anal sphincter defects with a maximum extension of up to 82°.

Grossi et al. did not include patients with internal or external anal sphincter defects greater than 60° or 90° when comparing the postoperative anal sphincter muscle contractility and tension in 10 patients after SphinKeeper® implantation to those receiving the predecessor (GateKeeper®) [17]. They observed that the SphinKeeper® led to higher muscular tension and similar morpho-functional remodelling of the anal sphincter complex as compared to the GateKeeper®.

The effect of prostheses movement on anal sphincter function remains controversial [18]. Noteworthy, even the exact definition of the type of dislocation varied among studies, making accurate comparison difficult. We have proposed a clear categorization to make future studies comparable [19]. Initially, Ratto et al. identified prostheses migration as an adverse event in one patient (10%), without leading to clinical consequences. In contrast, Grossi et al. found misplaced prostheses in 40% of included patients [17]. However, they did not categorize the patients according to success and prostheses displacement. Accordingly, the impact of dislocation on functional outcome was not discussed. Similarly, Litta et al. reported on 19 patients (45%) with insufficient prostheses localization [11]. The target area was defined at the level of the upper and middle thirds of the anal canal. Notably, FI showed a higher improvement in those patients who had an adequate placement of SphinKeeper® implants.

In a retrospective multicenter audit, Leo et al. reported that despite 10 inserted implants, ultrasonography assessment could only detect a median of seven prostheses after surgery [10], with only five of them were positioned correctly. However, clinical benefit was unrelated to the rate of misplaced/migrated implants.

In the present analysis, we demonstrated that prostheses dislocation affected later functional improvement negatively. Thus, an adequate number of well-sided prostheses are necessary to obtain good outcome, highlighting the impact of careful surgical implantation [20].

The indication for choosing the SphinKeeper® surgery has not been clearly defined so far. In the early studies of the predecessor (GateKeeper®), mainly patients with passive FI were selected for surgery [5, 12]. In the following years, the indications were extended, and patients with urge incontinence or incontinence to flatus were included for SphinKeeper® treatment too, resulting in moderate treatment success [10, 17].

We included patients with active and passive incontinence types and did not exclude patients with even severe forms of incontinence either. Patients with a higher degree of internal sphincter muscle damage seemed to particularly benefit more from the SphinKeeper® surgery.

This can be used as a new selection parameter in the context of daily clinical practice, which till now was not established.

In the beginning of the implementation of this operation, only patients with IAS and EAS sphincter lesions less than 60° were chosen for therapy [5, 10, 17]. SphinKeeper® implantation was later expanded to include patients with IAS or EAS defects reaching 120° [7, 9, 11]. Notably, the only trial that included individuals with defects greater than 120° still showed a significant reduction in the St. Mark’s incontinence score after SphinKeeper® implantation [8]. As a consequence, it can be speculated that childbirth trauma as well as patients with iatrogenic injuries of the internal sphincter muscle are good candidates for SphinKeeper® surgery.

Few limitations of the study need to be addressed. Despite the fact that this study included the second-largest sample with the longest follow-up period published to date, a higher number of patients are needed to obtain further evidence. In addition, patients with all types of incontinence were comprised, resulting in a heterogeneous group of incontinent patients. However, all data were collected prospectively, and patients were carefully assessed by standardized questionnaires and rigorous functional diagnostic work up.

## Conclusion

In our long-term study conducted over nearly 2 years, we observed that the SphinKeeper® procedure represents a safe option for patients with fecal incontinence (FI), resulting in a significant functional improvement in approximately 50% of the included patients. Interestingly, patients with a higher internal sphincter defect appeared to derive the most benefit from the SphinKeeper® operation, while the type and severity of FI had no impact on subsequent functional outcomes. However, it is worth noting that dislocation of the prostheses was associated with less favorable results.

## Data Availability

The raw datasets generated during and/or analyzed during the current study are not publicly available due to the sensitive nature of the questions asked in this study but are available from the corresponding author on reasonable request.
